# How Thermal Perceptual Schema Mediates Landscape Quality Evaluation and Activity Willingness

**DOI:** 10.3390/ijerph192013681

**Published:** 2022-10-21

**Authors:** Wenbo Li, Jiaqi Wu, Wenting Xu, Ye Zhong, Zhihao Wang

**Affiliations:** Urban and Landscape Design Lab, School of Art and Design, Hainan University, Haikou 570100, China

**Keywords:** thermal experience, thermal perceptual schema, landscape quality evaluation, activity willingness, landscape configuration

## Abstract

The use of outdoor space is closely related to local microclimate conditions. Some studies have shown that people form perceptual schemata based on their perceptual experience of microclimate conditions, which leads to perceptual bias, so it is necessary to further investigate how the thermal schemata formed by the accumulation of thermal experience affect the willingness to engage in activities, which will be beneficial to improve the use of urban space. Studies have not explored the relationship between the thermal perceptual schema (TPS), landscape quality evaluation (LQE), and activity willingness. Therefore, it is necessary to further investigate how thermal schemas formed by the accumulation of thermal experience affect activity willingness. A total of 3435 volunteers were surveyed online and divided into two groups, the first group for comfortable weather (*N* = 1773) and the second group for hot weather (*N* = 1662), and voted for each of the four dimensions of the five scenarios according to the TPS. This study found that socioeconomic status (SES) and age were the main factors contributing to TPS bias when perceiving the same destination according to TPS, and this difference was consistent in both groups, which affects the willingness to be active at the destination. The study also found that LQE may be a major factor in residents’ willingness to be active in more pleasant weather, while TPS plays a more important role in hot weather conditions. In addition, we investigated the relationship between TPS and residents’ activity willingness mediated by different landscape features and parameter configurations. These results indicate that the TPS formed by thermal experience accumulation affects people’s LQE and activity willingness, and that landscape configuration parameters play an important role.

## 1. Introduction

Multifactorial variables of the thermal environment outside buildings influence the activities of residents in outdoor public spaces [1,2]. In the past decades, a variety of thermal perception evaluation metrics have been constructed [3,4]. A growing number of studies have demonstrated the influence of physiological and psychological factors of outdoor thermal perception on human thermal perception [3,5], and some studies have made partial predictions of extrinsic perception through physiological metrics [6,7]; for example, using methods such as metabolic rate and body temperature to measure differences in thermal perception in different areas [8]. However, the traditional approach of treating neutral or acceptable temperatures as comfort conditions in this measurement process has also been criticized. Many studies have shown that people’s preferred temperatures differ significantly from neutral temperatures. It can be argued that the recognition of human perception in the current definition of thermal comfort has produced a significant shift in design goals. Some studies have suggested other factors that may influence thermal comfort, such as experience, expectations, the “naturalness” of the environment, and time of exposure, among others [9]. For example, dense vegetation and highly naturalized landscapes subconsciously create the three perceptual experiences of cool and comfortable microclimate, attractive, and least safe [10,11], and semi-enclosed spaces are often perceived to have higher comfort levels in terms of wind and sunlight [12]. Current research on thermal perception focuses on the physiological thermal responses of people in a space, ignoring the perceptual schema that people tend to form based on their perceived experience of these environments when making activity site choices. Some studies have also proposed to study thermal diversity in depth, where one of the essential parameters to be considered is the perception of thermal diversity of urban environmental attributes by open space users [13], which will better predict the activities they will perform and, therefore, it is crucial to understand the impact of TPS on site usage.

Generally, people make decisions about going out based on their perception of the destination, rather than reality [14,15]. Although recent studies have incorporated psychological and physiological influences into the modeling of thermal perception [16], relevant studies supporting the idea that landscape configuration and thermal experience factors influence activity intentions seem to have been overlooked so far. Therefore, it is necessary to understand and evaluate the impact of spatial configurations on people’s psychological perception of thermal, detached from physical indicators, whether their mental thermal changes if they lose specific social or physical characteristics, and whether imagined thermal affects the attractiveness of the destination so that it can be integrated into the design process.

## 2. Literature Review

### 2.1. Spatial Features and TPS

Researchers have studied whether spatial configuration parameters and scene characteristics influence psychological thermal perceptions. Lenzholzer et al. found, through interviews and measurements of site microclimate, that spatial configuration and proportions can act as visual cues to microclimate to suggest psychological thermal perception [12]. Additionally, a related study confirmed that visual factors, as one of the non-thermal factors, significantly confounded subjective thermal perception. Rosso et al. found that different pavement materials induced changes in psychological thermal perception through visuals and that places with higher green exposure show more positive thermal perceptions than places with higher exposure to buildings and sky at the same level of thermal climate index [17,18]. These related studies demonstrate that the physical spatial environment can influence thermal perception through previous perceptual experiences with visual cues. Moreover, people can be aware of the microclimate conditions of a given location based on experience [19], and it can be said that different landscape features of the environment can bring about different thermal perceptions in thermal memory and form perceptual schemata in the human mind [20]. This means that people’s choice of site is influenced not only by the thermal comfort of the environment, but also by the “landscape cues” that the configuration of the structured space brings to the residents’ psyche [21]. Although the above findings point out that these spatial configurations and features can influence people’s thermal perception of the environment through visual and perceptual means, and form long-term perceptual schemas, there is no specific research on whether TPS can affect people’s activity willingness towards a particular location.

### 2.2. Thermal Perception, LQE and Activity Willingness

Several studies have shown that thermal perception relates to the environment’s naturalness, aesthetic appreciation, and positive experience. Anavvi and Rajasekar found that satisfaction with aesthetic landscape elements influences thermal perception [22]. A 2021 study found that respondents who were dissatisfied with aesthetic qualities tended to report warmer thermal comfort votes, and vice versa [23]. For example, a range of design attributes, such as the proportion of spatial configurations, perceived color of surface materials, luminous environment, and satisfaction with landscape elements, statistically influenced votes for thermal perception [24,25]. This highlights the role of design elements in shaping thermal environments and suggests the need to consider the subjectivity associated with thermal perception in such outdoor environments. Additionally, several perception studies include a suitable microclimate as one of the criteria for judging site comfort, in addition to studies that show that site selection may also be based on amenities; for example, tennis courts in sports parks do not have a comfortable thermal environment, but still attract many people to participate. Thus, it can be seen that thermal environment factors or LQE alone do not fully explain the effect of the thermal environment on willingness to use public space [26].

### 2.3. Relationship between Thermal Perception and TPS

The characteristics of spatial impression cognition mainly emphasize a mental course of action on environmental information, and the constant repetition of this cognitive course of action forms fixed long-term perceptions [27,28]. For example, accumulated weather experience can lead to long-term perceptions that result in habitual judgments of instantaneous thermal perceptions of microclimatic environments [19,29], which dominate people’s behaviors and acceptance of sites [30]. Extensive research on schemas has shown that human behavior, including avoidance or preference for locations and events, is guided to some extent by schemas. In addition, studies have shown that individual identity and growth history significantly impact environmental behavior and thermal perception [31,32]; for example, women tend to be less tolerant of hot environments than men. Jiaqi Niu et al. found that physical factors and thermal perceptions of green had an impact on attendance, duration, metabolic rate, and frequency of physical activity, with middle-aged participants and older adults having lower thermal perception compared to younger participants, which made them more willing to be active in green areas. Younger people, and those with higher socioeconomic status, tend to live in indoor environments with cooling facilities and are more sensitive to high outdoor temperatures.

Some studies have also found significant differences in the neutral temperature of people living in different climatic zones [33]. However, people are highly adaptive to thermal environments; indoor and outdoor climatic environments at different time scales can shape people’s past thermal experiences [8,34]. Long-term thermal experiences can lead to different thermal expectations of future environments [6]. There is a high correlation between thermal environments and activity in outdoor spaces and the locations where they gather, which is also influenced by the background climate and people’s experiences and behavioral adaptations [26].

The above studies illustrate that people’s long-term immersion in designated microclimatic environments affects individuals’ perceptions and behaviors, resulting in the formation of perceptual schemata through long-term experience perceptions, i.e., long-term memory from mental adaptation.

### 2.4. Research Questions

As mentioned above, although studies have shown that activity intentions are essentially a result of perceptions of destination history, much of the existing research on thermal diversity has focused on the thermal perceptions of people in their environment. While a small body of literature suggests that TPS can lead to thermal perception bias, the impact of TPS on destination activity intentions and LQE is unclear. The latter is crucial for the study of sites, allowing for more effective shaping and use of community spaces and other urban spaces. Based on the above, the focus of the research in this paper is as follows:

(1) Whether TPS varies across populations.
(2) To verify the role of TPS in the relationship between LQE and activity intentions.
(3) To analyze the role of landscape parameter configuration on TPS, LQE, and activity willingness.

## 3. Methods

### 3.1. Experimental Materials and Scene Simulation

Approximately 80% of human perception originates from vision [25], a powerful sense for perceiving outdoor thermal environments [34]. Existing studies have demonstrated that long-term thermal perception can build visual representations of stimulus objects in the form of features [35]. Moreover, the use of vision to study perception as an assessment method has been used in different fields to establish methodologies that have been combined in various outdoor thermal perception studies. For example, Lenzholzer et al. explored the similarities and differences between microclimate schemas in people’s minds and microclimates in real environments by asking subjects about microclimates in a given location [12]. Cortesão et al. tested residents’ subjective perceptions of the thermal environment of a site using individual landscape elements from photographic images as visual symbols [36]. Using visual methods to assess thermal perception as a basis, to some extent, thermal perception can explain thermal environment depictions containing apparent landscape configurations, leading to further discussion of how TPS relates to landscape configurations [37].

Numerous studies have shown that many uncontrollable factors affect the real world, such as light intensity [38], noise [39], and material glare [40], which may lead to biased experimental results. In contrast, using virtual images instead of natural landscape scenes has good reliability [41,42,43,44]. It can effectively control the light, perspective, and color of photographs [45], and it is widely used in studies related to landscape perception [46,47,48,49]. To test the differences in TPS of residents in different regions and ensure fairness, the locations were virtual. The questionnaire was designed without images with significant geographical factors; the simulated images were representative of real-world features. This paper focuses on the effect that visual characteristics of TPS, LQE, and configuration parameters of the landscape can have on willingness to move, so other aspects of the landscape experience, such as physiological temperature, sound, and smell, are not included in the study.

The reason for choosing the public space attached to the old residential communities as the research site is because this public space is usually more closely related to the residents. Because of its proximity to their homes and accessibility, it is generally used by the community’s long-term residents [50,51,52], so it is a more suitable object than squares and parks. Before obtaining the data, objective, systematic, and quantitative data collection was carried out using content analysis to present photographs of sites that were more familiar to the interviewees [53]. The keywords of “community leisure”, “community public space”, and “community activity space” were searched in the public information platform, and 3162 photos with standard features were finally collected for multi-feature classification. After data screening and removing meaningless and repetitive images, 921 images could be used for image feature analysis, followed by image feature coding, conducted manually by the researcher based on image information. The specific steps refer to similar studies by Agapito et al. and Lv et al. [54,55]. It was stipulated that the coding increases by 1 for each independent landscape feature. To solve the reliability problem, the two researchers’ coding process was conducted independently, 92% of which matched each other, and the images that did not match were deleted. The typical features of the final screened images presenting mainly public spaces are shown in Table 1.

**Figure 1:**
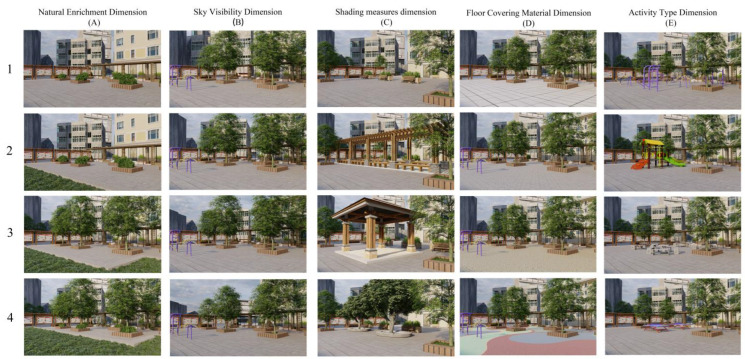
Stimulus material design for different landscape parameter configurations used for the experiment.

Scenes of the built environment, plant combinations, and event facilities within site were created based on the analysis of the common characteristics using the 3D modeling software SketchUp 2018, with the simulated picture viewpoint set at a height of 160 cm and a 28 mm focal length used for the field of view, which was verified to be a valid measure of respondents’ impressions of the amount of greenery when viewed from a fixed position (Figure 1) [56]. The variables controlled in the figure follow the environmental psychology definition of spatial connotation combined with previous field-based studies by scholars. The factors that would form TPS were selected: naturalness (green diversity) [9], physical (form and space) [12], functional (activity) [57], psychological (meaning people give to the place) [58], and material (ground cover) [59], aspects of spatial parameters that could affect TPS. The whole experiment was designed with five variables; each variable controlled in the photo was divided into different characteristics and dimensions. In order to prove the validity of the material, a pretest was conducted.

### 3.2. Pretest

The pretest was recruited online, and the final sample consisted of 87 participants (51.7% female, Mage = 23.6) after excluding invalid questionnaires. The purpose of the pretest was to test the validity of the experimental material, and the questionnaire was set up to simulate pictures to assess participants’ familiarity with the space, imaginable perceptions, and the rationality of the questionnaire questions, in which the A1 dimension of natural richness was used to simulate pictures to investigate the scene with three questions: “Are you familiar with the environment of the displayed pictures??” (1 = very unfamiliar, 7 = very familiar) “Can you imagine the cold/hot situation of the location based on the pictures?” (1 = hard to imagine, 7 = easy to imagine). The results showed that the mean value of the simulated pictures was significantly higher than 4, indicating that the simulated pictures could represent the space in real-life well (M = 5.57, SD = 1.263,T = 11.626, *p* < 0.001), and the participants’ perceptibility of the simulated pictures was significantly higher than 4, indicating that most participants can perceive the hot/cold of the site through imagination (M = 5.34, SD = 0.950, T = 13.200, *p* < 0.001), which proved the validity of the experimental material.

Next, we tested the reasonableness of the questionnaire [60,61], referring to existing LQE studies, and there was a high level of agreement between the questions “The landscape of my residential environment is very beautiful” and “In the landscape of my residential environment, I find many places very beautiful”. Therefore, we modified the reference scale and added relevant questions affecting the LQE (Table 2). The Cronbach’s alpha for the four modified LQE scales in the pretest was 0.828, with high internal reliability.

### 3.3. Experimental Settings and Participants

The experiment recruited volunteers through the internet, and a total of 3435 volunteers were successfully recruited and divided into two groups (the first group was the comfortable weather group, and the second group was the hot weather group). All questionnaires were returned within three days, and each volunteer was given a reward at the end of answering the questions. The whole experiment was designed with four parts: (1) The first part was the respondent demographic characteristics. (2) The second part was the TPSV statistics, which divided the participants into two groups. Both groups used pictures of the same 15 scenarios, which the first group presented the participants with the following instructions: “The weather today is neither windy nor rainy; you have to go out and hang out during the day on weekends; this place in the community is very close to your house, will be going to this venue in a while”. The second group modified the introduction description: “It’s hot today, you still decide to go out for a while, this place in the community is close to your house, and you will go to this venue in a while.” Respondents voted on the simulated pictures according to the TPS, using a Likert scale of 1–7 to assess the local area; where 1 means “very cold” and 7 means “very hot”. To ensure accuracy, one participant was shown only one image. (3) To test whether TPS would affect the LQE and willingness to engage in activities at the site, the arithmetic mean of the responses to the four statements under LQE was used as an indicator value for the subsequent analysis [60], followed by asking about their willingness to engage in activities in the scenario, in addition to which we added some questions to the questionnaire that were not related to the purpose of the study to make the meaning of the study more ambiguous.

### 3.4. Data Analysis

The entire experimental data were statistically analyzed using SPSS 26.0 software. First, each variable was divided by regrouping (Table 3). Differential analysis of demographic variables on TPS was conducted using binary logistic regression.

In addition, the collected data were categorized by residents living in the south or north, and to control for transient populations (such as short-term renters), those who had lived there for more than 5 years were analyzed as a sample to test as to whether long-term thermal history affects TPS versus overall perception. Socioeconomic status (SES) was used as a test for differences in the effect of living standards on TPS, including income, educational background, and type of occupation (data were extracted from the questionnaire), and was calculated as:

SES = (β1 × Z_e_ + β2 × Z_O_ + β3 × Z_i_)*/*εf.

where β1, β2, and β3 are the factor loadings for extracting the relevant question items; ZE, ZO, and ZI are the standard scores of the variables related to educational background, occupation type, and monthly income, respectively; and εf is the initial eigenvalue variance percentage of the first factor.

Then we investigated whether different landscape parameters and environmental features affected TPS. ANOVA was used to test whether landscape parameters and environmental features lead to significant differences between TPS, LQE, and activity willingness, and an LSD test was used to further determine the differences between different landscape classes to provide a basis for subsequent studies.

The current quantitative evaluation of landscape quality using semantic segmentation of images with deep learning has been widely used in urban streetscape research, such as urban imagery perception, the impact of street greening on residents’ walking behavior, measuring street space quality, and urban greenway planning [62,63]. Using an artificial intelligence convolutional neural network (CNN) for scene element recognition, the bounding boxes of landscape elements (such as sky, trees, people, ground, etc.) are output and returned with detailed area share calculations (Table 4). Based on image recognition, analysis was performed using Spearman’s rank correlation with stepwise multiple linear regression. First, each landscape parameter was used as an input variable and residents’ perception parameters as output variables to further calculate the correlation between the area share of each landscape element within the images and the public TPSV and LQE with the willingness to move around the site. Afterwards, the two groups of returned landscape element percentages were linearly regressed against TPS, LQE, and activity willingness, respectively, to explore the significant drivers of landscape parameter configuration on TPS, LQE, and activity willingness.

Finally, to test the mediating role of landscape configuration in the middle of TPS and LQE in activity willingness, using SPSS 26.0 with PROCESS plugin version 3.5, following Hayes proposed method, using α = 0.05 as the significance level, 5000 Bootstrap sample size simulations were conducted at 95% confidence intervals. The indirect effect (IE) and indirect effect quantity (IEQ) were separately reported.

## 4. Results

### 4.1. Reliability

The Cronbach’s Alpha coefficient was used to test the interclass reliability of the LQE scale. The Cronbach’s Alpha coefficient for the LQE calculated for the Comfortable Weather group was 0.880, with a range of alpha from 0.843 to 0.851 for the four items, and the Cronbach’s Alpha coefficient for the LQE for the Comfortable Weather group was 0.880, with a range of alpha from 0.805 to 0.848 for the four items. The Cronbach’s Alpha for both groups was more significant than 0.801, with good internal reliability [64].

### 4.2. Description of Findings on Demographic Variables

As shown in Table 5, the questionnaire collected basic information about the respondents, including gender, age, length of residence in the current community, income, educational background, frequency of visits and length of stay in public spaces, and whether they live in a southern or northern city.

Among the samples returned for Group 1, the sample size was larger in southern cities than in northern cities (58.4% in southern cities, compared to 41.6% in northern cities) [65], slightly more females than males (51.8% females), a smaller sample size of fewer than 15 years old (2.4%), and 32.8% of participants had lived in the neighborhood for 5 to 10 years. Most subjects were students or engaged in intellectual work. Their educational background was primarily concentrated at university level or above (43.9%), and 36.7% of the participants had an income of less than 3000 RMB. Most residents’ outdoor activities were mainly concentrated around 1–2 times a week (34.3%), with the subsequent most frequent participation 3–4 times a week (31%), and half of the residents concentrated between 16 and 60 min per activity (51.4%).

The sample in Group 2 was more evenly distributed between males and females (49.2% female), with the majority of ages between 16 and 25 (30.1%) and 26 and 35 (29.2%), and more than 51.6% had a university degree. More than 60% of the participants had lived in their current neighborhood for more than five years, with a smaller sample of 55 cases (3.3%) who were retired, most of the income was below 7000 RMB (69.6%), and 46.5% of the residents were in public spaces more than three times a week, with most lasting less than 60 min (88%).

### 4.3. Demographic Characteristics Associated with TPS in Two Groups

The estimated coefficients and odds ratios of the statistical correlation model between TPS and demographic variables are shown in Table 6. After accounting for significant correlates, the binary logistic LR stepwise regression model showed that age and living standard would lead to significant differences in the evaluation of TPS, consistent under both weather scenarios. The Hosmer-Lemeshow goodness-of-fit test values for both groups were >0.05, indicating that the model’s predicted values were accurate with the observed values.

Between the three groups by age, in Group 1, residents aged 26 to 35 years did not significantly differ from residents aged 25 years with respect to TPSV at the exact location (b = 0.078, *p* = 0.657, OR = 1.081,95% CI = 0.765 to 1.528); residents aged 35 years and older tended to perceive the exact location as more equipped with cooler climatic conditions (B = −1.323, *p* < 0.001, OR = 0.266, 95% CI = 0.200–0.354); and residents between the ages of 25 and 35 years tended to perceive the exact locations as having hotter microclimatic conditions compared to residents over the age of 35 years (B = 1.402, *p* < 0.001, OR = 4.062, 95% CI = 2.965~5.565). The variability in living conditions was mainly manifested by the fact that residents with higher living conditions perceived the destination as hotter (B = 0.579, *p* < 0.001, OR = 1.784, 95% CI = 1.388–2.293).

In Group 2, perceived as hot weather, the difference was significant for residents in the age group 26 to 35 years or older compared to those under 25 years (B = −1.111, *p* < 0.001, OR = 0.329, 95% CI = 0.218–0.498), while TPSV for residents over 36 years vs. those under 15 years was similar to the sample 1 group, considering the microclimate in the same location conditions were cooler (B = −0.672, *p* < 0.001, OR = 0.188, 95% CI = 0.128–0.275). Residents between the ages of 26 and 35 tended to give higher TPSV ratings than residents over 35 years (B = −0.672, *p* < 0.001, OR = 0.188, 95% CI = 0.128–0.275). Additionally, the effect of SES on TPS remains significant in Group 2, with higher living conditions tending to influence higher TPSV scores. (B = 0.854, *p* < 0.001, OR = 2.349, 95% CI = 2.005–2.752).

In summary, the results of the TPS analysis in the two sample groups indicate that different age groups lead to TPS deviations for the same sites, and that those with higher ages typically perceive the same destinations as not as hot compared to the other two groups. In addition, the effect of SES in both groups would increase TPSV by a factor of 1.581 versus 2.349 per quartile unit, respectively, with higher socioeconomic status residents tending to perceive a hotter TPS for the destination.

### 4.4. How Will TPS, LQE, and Activity Willingness Relate to Landscape Configuration Parameters and Site Characteristics?

ANOVA was used to analyze the significance of the means of TPSV, LQE, and activity willingness between the four dimensions in five scenarios under two weather conditions in this study. The groups with significant differences were analyzed by LSD post hoc two-comparison test results (Table 7 and Table 8).

In both samples, for the four different levels of greenery, richer greenery tended to mean cooler in the TPS, which was consistent and significantly different in both groups; the LQE of the site also increased with the increase of greenery, indicating that natural vegetation in the community public space increased the LQE and activity willingness of residents. However, overly natural vegetation led to a decrease in activity willingness. However, in hot weather, the activity willingness of residents was significantly different, Nevertheless, in contrast to the trend of comfortable weather, the activity willingness increased with the rise of greenery level.

The TPSV of the sky dimension B1 scenes was significantly lower in the comfortable weather group than in the other groups, suggesting that the proportion of sky may be positively correlated with TPS, and that it is accompanied by a gradual increase in residents’ LQE and activity willingness. The TPSV of residents in the hot weather group still increased along with the increase in the percentage of the sky, while LQE and activity willingness tended to decrease.

There is a significant difference in the perceived mean values of TPSV, LQE, and activity willingness between the two groups in the choice of shading methods, and the TPSV, LQE, and activity willingness of the C1 dimension without shading facilities are not very positive in each group compared to the C2, C3, and C4 dimensions. In terms of the use of three different types of shade—pavilion, landscape corridors, and trees—the site characteristics of the pavilion and high-canopy trees create a cooler feeling in residents’ consciousness. The use of landscape corridors tends to have higher LQE ratings and activity intentions compared to the two common elements of pavilion and landscape corridors in old residential communities, when the weather is more comfortable. However, when residents perceived the outdoors as hotter, the two different shade facilities did not significantly differ in LQE and activity willingness.

Regarding paving materials in Group 1, the floor with concrete paving had the highest TPSV rating. Its LQE rating was significantly different compared to the other three materials groups and had a negative relationship with willingness to move. In contrast, in the group with hot weather, the four groups did not show significant differences in TPSV, but, scenarios using concrete paving materials still have the highest TPSV, lowest LQE and willingness to move.

Moreover, there were also significant differences in terms of activity type. The TPSV was significantly higher for the common types of outdoor children’s outdoor activities than for the other two groups in the case of comfortable weather. There was no significant difference between the four experimental materials in hot weather; in terms of LQE, the E4 sites with higher intensity of movement were significantly higher than the other three groups, but there was no significant difference between the activity site types in hot weather.

**Figure 2:**
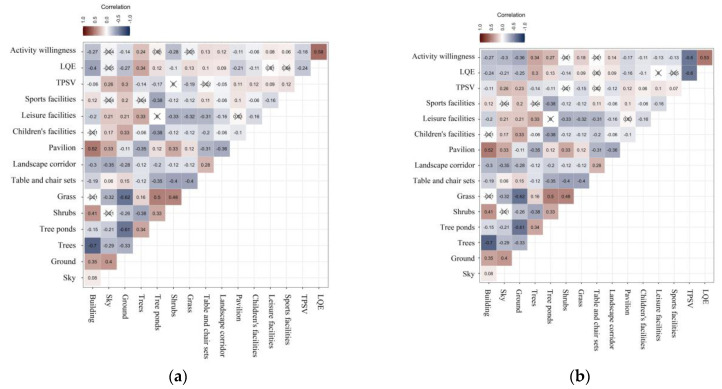
(**a**) Spearman’s rank correlation matrix between the percentage of landscape parameters (%) and TPSV in Group 1; (**b**) Spearman’s rank correlation matrix between the percentage of landscape parameters (%) and TPSV in Group 2.

Figure 2 shows the correlations between the proportions of landscape parameter factors and PTSV, LQE, and activity willingness (the paving approach in the experimental material involved the influence of different materials, so we excluded the correlation data for the four dimensions in this stimulus material in the following analysis). Correlation analysis showed that TPSV in Group 1 increased with the proportion of sky, ground, landscape corridor, and the three site activity types; whereas it decreased with the increase of trees, tree pools, grass, and pavilions. The increase in perimeter-enclosed buildings and excessive exposed ground in outdoor public spaces will lead to a decrease in LQE and activity willingness. In addition, the rise in the proportion of shrubs and landscape corridors may be a reason for the reduction of LQE and activity willingness. Trees, tree ponds, tables, and chairs, pavilions, leisure activity facilities, and sports facilities showed a weak positive correlation with activity intention. Additionally, in the second group of hot weather, the LQE of the sky changed from insignificant to a negative correlation with activity intention. The relationship between shrubs, grass, recreational facilities, and sports facilities with LQE and activity intention changed from a positive to a negative correlation.

The correlation analysis only illustrated the relationship between the TPSV of individual landscape objects and LQE and activity willingness, and did not consider the problem of multicollinearity. Therefore, controlling for the influence of variables accounted by other observational parameters on this basis, a stepwise multiple linear regression was used to calculate standardized regression coefficients for landscape configurations with adjusted R^2^ values (VIF < 2), which indicate the percentage of variance explained by all visual features in the landscape configuration, as well as partial R^2^ values.

After adding all relevant control variables (Table 9), the proportion of landscape configurations in Group 1 explained approximately 16.5% of the variance consistency of TPSV (F = 41.896, *p* < 0.001, Adj.R^2^ = 0.165). The proportions of the sky (b = 0.165, R^2^_partial_ =0.1168, *p* < 0.001), ground (b = 0.256, R^2^_partial_= 0.232, *p* < 0.001), tree pools (b = 0.077, R^2^_partial_ = 0.071, *p* < 0.01), children’s facilities (b = 0.079, R^2^_partial_ = 0.080, *p* < 0.01), and sports facilities (b = 0.078, R^2^_partial_ = 0.083, *p* < 0.01) were positively and significantly associated with TPSV. Trees (b = −0.157, R^2^_partial_= −0.159, *p* < 0.001) and corridors (b = −0.058, R^2^_partial_ = 0.056, *p* < 0.05) were significantly and negatively associated with TPSV.

The landscape configuration accounted for approximately 21.1% of the LQE, where sky (b = 0.129, R2partial = 0.108, *p* < 0.001), trees (b = 0.338, R^2^_partial_ = 0.271, *p* < 0.001), shrubs (b = 0.145, R^2^_partial_ = 0.101, *p* < 0.001), and sports facilities (b = 0.090,R^2^_partial_ = 0.096, *p* < 0.01) were the main factors positively affecting LQE; buildings (b = −0.197, R^2^_partial_ = 0.090, *p* < 0.01), ground (b = −0.171, R^2^_partial_ = −0.131, *p* < 0.001), children’s activity facilities (b = −0.058, R^2^_partial_ = 0.062, *p* < 0.05), and the increase in the percentage of LQE were the main factors negatively affecting LQE.

The configuration of landscape parameters could explain about 15.8% of the willingness to move around the site, with sky (b = 0.179, R^2^_partial_ = 0.124, *p* < 0.001), trees (b = 0.251, R^2^_partial_ = 0.093, *p* < 0.001), tree ponds (b = 0.131, R^2^_partial_ = −0.111, *p* < 0.001), sports facilities (b = 0.115, R^2^_partial_ = 0.113, *p* < 0.001), and pavilions (b = 0.115, R^2^_partial_ = 0.117, *p* < 0.001), and an increase in the proportion of the site can increase the willingness to move around; buildings (b = −0.169, R^2^_partial_ = 0.063, *p* < 0.01), ground (b = −0.124, R^2^_partial_ = 0.162, *p* < 0.001), and shrubs (b = −0.278, R^2^_partial_ = 0.163, *p* < 0.001) reduce the activity willingness of the site.

In Group 2 the landscape configuration explained approximately 11.7% of the variance consistency of TPSV (Table 10); consistent with Group 1, the visible ratio of sky (b = −0.231, R^2^_partial_ = −0.208, *p* < 0.001) to ground (b = −0.231, R^2^_partial_ = −0.208, *p* < 0.001) was positively associated with TPSV, and the negative effect of trees (b = −0.231, R^2^_partial_ = −0.208, *p* < 0.001) with TPSV remained significant.

Landscape configuration explains 16.1% of LQE, sky (b = −0.123, R^2^_partial_ = −0.127, *p* < 0.001), ground (b = −0.123, R^2^_partial_ = −0.127, *p* < 0.001), and children’s facilities (b = −0.158, R^2^_partial_ = −0.220, *p* < 0.001) had a significant negative main effect on LQE, and trees (b = 0.308, R^2^_partial_ = 0.318, *p* < 0.001) remained the main factor that positively influenced residents’ landscape quality evaluation; landscape configuration parameters explained about 10.8% of activity intentions, with trees (b = 0.320, R^2^_partial_ = 0.204, *p* < 0.001), shrubs (b = 0.115, R^2^_partial_ = 0.070, *p* < 0.001), tree ponds (b = 0.155, R^2^_partial_ = 0.136, *p* < 0.001) and pavilions (b = 0.109, R^2^_partial_ = 0.098, *p* < 0.001) having a significant positive main effect on LQE; sky (b = −0.148, R^2^_partial_ = −0.155, *p* < 0.001) versus ground (b = 0.136, R^2^_partial_ = 0.113, *p* < 0.001) share had a significant negative main effect on activity willingness in hot weather.

### 4.5. How Will TPS, LQE, and Activity Willingness Relate to Landscape Configuration Parameters and Site Characteristics?

In the above study, both groups positively correlated TPS and LQE with activity willingness. To test the relationship between TPS and LQE and activity willingness, we controlled for the effect of LQE on activity willingness and performed a multiple linear regression. For each model, standardized regression coefficients were calculated (Table 11). Both groups had significant effects on activity willingness and LQE, with a strong negative relationship between TPS and activity willingness in Group 1 [(b = −0.279, F = 122.221, *p* < 0.001, R^2^ = 0.077, 95% CI = −0.296–0.207), (b = −0.226, F = 76.879, *p* < 0.001, R^2^ = 0.050, 95% CI = −0.281–0.178)], and a significantly weaker relationship between TPS and LQE and activity willingness after controlling for the effect of LQE [(b = −0.160, F = 395.709, *p* < 0.001,R^2^_partial_ = −0.132, 95% CI = −0.182–0.105), (b = −0.069, F = 362.548, *p* < 0.01, R^2^_partial_ = −0.081, 95% CI = −0.115 to−0.025)].

In Group 2, there was a negative relationship between TPS, LQE, and activity willingness [(b = −0.643, F = 935.428, *p* < 0.001, R^2^ = 0.413, 95% CI = −0.653~−0.574), (b = −0.646, F = 949.206, *p* < 0.001, R^2^ = 0.417, 95% CI = −0.762 to −0.671)], and after controlling for variables, the correlation coefficients between TPSV and LQE and activity willingness decreased, but there was still a strong relationship relative to Group 1 [(b = −0.467, F = 557.272, *p* < 0.001, R^2^_partial_ = −0.436, 95% CI = −0.496–0.396), (b = 0.467, F = 557.272, *p* < 0.001, R^2^_partial_ = −0.436, 95% CI= −0.496–0.396)].

The above regression analysis showed a significant correlation between TPSV of the two types of weather and LQE and activity willingness, respectively. Therefore, considering that TPS may be an essential factor affecting LQE and venue activity willingness, a mediating effect analysis of the relationship between the three was conducted to test a hypothesis.

**Figure 3:**
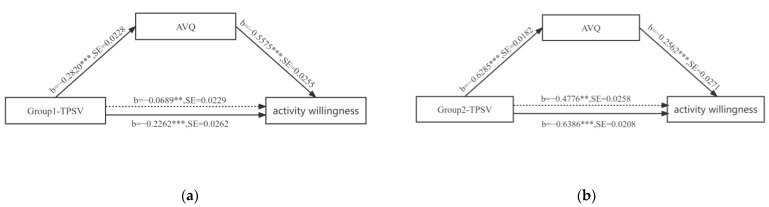
Mediating effect between TPS and LQE’s activity willingness in Group 1 (b is the standardized regression coefficient; SE is the standard error). (**a**) Mediating effect of LQE between TPS and activity willingness in Group 1; (**b**) Mediating effect of LQE between TPS and activity willingness in Group 2 (b is the standardized regression coefficient; SE is the standard error). (Note: *** *p* ≤ 0.001, ** *p* ≤ 0.01).

Using the bias-corrected percentile Bootstrap method (5000 repetitions were taken), the results of the correlation analysis in both groups showed that the bias-corrected confidence interval did not include 0, so this relationship did exist (Figure 3 and Table 12). The higher the TPSV rating at the site, the lower the willingness to engage in activity at the site, and the analysis of the subsequent paths showed that the TPSV at the site increased, leading to a lower landscape quality rating. At the same time, the more LQE decreased, the lower the activity willingness, and the two data groups showed the same trend. However, when controlling for LQE as a mediating variable, the correlation between TPSV and activity willingness in Group 1 remains significant but weaker, while TPSV and activity willingness in Group 2 remains significant and has a strong correlation.

### 4.6. Mediating Effects between Landscape Parameters and TPS, LQE, and Activity Willingness

The above study found a significant correlation between TPS and activity willingness in two different groups of contexts, and some landscape parameters were also found to be significantly correlated with TPS, LQE, and activity willingness, so landscape parameter configurations may be the basis for linking TPS, LQE, and activity willingness. A subsequent 39-item (13 landscape configurations * three mediated paths) mediation analysis was conducted for each of the two data sets to test this hypothesis. The mediation model with significant effects was reported (Table 13).

## 5. Discussion

### 5.1. Demographic Variables and TPS

First, this study verified whether TPS is influenced by thermal experiences, and thus, differences in the perception of destinations. It was determined that different age groups and socioeconomic statuses lead to this perception bias, and that there are significant differences in both groups.

The difference in the effect of age on TPS is mainly reflected in the fact that older residents tend to perceive the exact locations as cooler compared to the rest of the population. This bias may arise because older adults have lower activity levels, which means a lower metabolic rate, and typically require higher ambient temperatures than younger people to feel comfortable [66]. Furthermore, in a 2019 study, it was noted that despite reduced thermoregulatory capacity [67], older adults typically perceive lower thermal risk, leading to reduced use of coping behaviors, so significant differences in age then likely shape thermal schema through the process of physiological habituation to environmental familiarity [68]. As a result of this perceptual bias, older adults likely misjudge their choice of activity site, and this perceptual bias poses a potential health risk to older adults in hot weather. Furthermore, SES leads to a change in TPS, which tends to be higher for residents with a higher standard of living. Studies have shown that highly educated individuals spend more time working indoors, have less access to green spaces, are increasingly “picky” about thermal environments due to past habituation in more comfortable thermal environments, and differ in thermal comfort levels between outdoor workers and government employees, showing differences in adaptation and expectations. Furthermore, a meta-analysis of thermal mitigation strategies found that over-reliance on air conditioning and avoidance of high heat stress conditions in high-income individuals may diminish the thermoregulatory benefits of thermal acclimation [69], and this formation of psychological schema is similar to that found in many studies of thermal perception [70].

Elderly and low-income people or tenants tend to avoid air conditioning due to the cost of cooling appliances [71,72], their physiological response to cold temperatures, and the structure of their housing units. A study that included 1000 seniors found that most older respondents adopted mostly adaptive behavior in hot conditions due to financial concerns, such as finding cooler resting places, wearing less clothing, and drinking more water [73], rather than cooling down through coolers such as air conditioners [74,75]. In conclusion, it is easy to see those older adults mainly obtain a more comfortable environment in two ways: adaptive behavior or adjusting to the thermal environment. Some recent studies have proposed methods to increase activity willingness, such as installing additional pools, remodeling green roofs, or shifting activity spaces [76,77]. However, these methods may not apply to resident subjects with limited funds or urban spaces with limited activities; but, many studies in recent years have claimed that public spaces in cities may provide an alternative solution by visiting parks as a passive cooling strategy that evokes adaptive behavior in older adults with low SES levels [78]. Therefore, exploring the differences between TPS is essential for urban renewal and increasing the utilization of old residential community space. Still, it also provides a new direction for the construction of daily activity spaces for specific groups of people.

### 5.2. Relationship between TPS, LQE, and Activity Willingness

The findings from this study provide crucial information about the willingness to enhance activity in different weather conditions. The first thing that can be determined is that in two types of weather, residents influence the willingness to engage in activities based on the TPS that has been generated (Group1-TPSV, b = −0.069, R^2^_partial_ = −0.081), (Group2-TPSV, b = −0.472, R^2^_partial_ = −0.441), which implies that residents can have an influence on location activity by generated TPS, but complex relationships may emerge when other influences are involved. For example, the correlation analysis between TPSV, LQE, and activity willingness showed that the correlation between TPSV and activity willingness significantly decreased when LQE was included as a control variable, and this decrease in the relationship suggests that people are more inclined to consider LQE as the main factor of activity willingness in average weather. However, when in hot weather, often the local microclimate becomes the main factor influencing the willingness to be active. Moreover, in this study, it is similar to the findings of Kevin Ka-Lun Lau et al. in the sense that people usually give lower LQE ratings when they are dissatisfied with the microclimate of the destination, and it affects activity willingness, and this chain relationship works more clearly in hot weather. Therefore, further work is needed in cool environments to determine the environmental quality and characteristics, such as avoidance of architectural shading, rich vegetation, and good sky visibility, to provide a better outdoor environment in high-density urban environments to enhance residents’ willingness to move around. The creation of public spaces in hot climates, on the other hand, is based more on the TPS of residents to enhance their willingness to move around. The aesthetic quality of outdoor spaces is highly correlated with thermal comfort, and plays a vital role in how people perceive the thermal comfort of a site.

### 5.3. Relationship between Landscape Configuration in TPS, LQE, and Activity Willingness

TPS is not always negatively correlated with activity intention and LQE, where landscape configuration parameters play a complex role. For example, along with an increase in the proportion of greenery, both TPSV and LQE of the site appear to present a more idealized situation, but the activity intentions of residents decrease at high levels of vegetation, and these results are not surprising; people generally have a high preference for diverse plantings and can predict landscape preferences well [79,80], as has been widely evidenced in previous studies [81,82]. It has also been noted that aesthetic quality and activity preference differ, as the former focuses on viewing appreciation and the latter implies participation [83]. Although the above studies pointed out that different seasonal changes would cause changes in aesthetic preferences and activity intentions, the role of TPS in this was ignored, and only environmental changes were explained. Based on this study, this paper found that when the microclimate of the destination was in a hot state, residents would still have higher activity intentions for high-density greenery, which was the opposite of the activity intentions that appeared in Group 1. In addition, the study found that regardless of the weather, trees and grass created a cooler perceptual icon in the TPS of residents, and increased activity intentions. However, not all types of vegetation helped optimize TPS, and thus, enhanced activity intentions; for example, the correlated mediating path of TPSV → shrubs → activity willingness was not significant in both groups. This can be explained by Appleton’s foreground-shelter theory [84], where the foreground is one of the features conducive to survival, which can be explained by the fact that too many shrubs increase the perceived insecurity of the environment while weakening the willingness to move, which is why shrubs in the scene remain unpopular despite the partial shade and shade they can provide.

Previous studies have shown that people generally prefer sky views [85]. When the weather is hot, the increase in the percentage of the sky is often accompanied by a decrease in the willingness to be active. This can be explained using the findings from the above study: TPS was the main predictor of activity willingness in hot weather, and similar studies have explained that people prefer shade to reduce solar radiation in hot summers. In addition, a previous study showed that a certain level of sky view does not reduce thermal comfort and is necessary to improve outdoor comfort, so appropriate sky views should be retained even in hot environments [86]. Next, different site attributes affect residents’ LQE and activity preferences. Previous studies on landscape environment preferences are usually based on environments with high mobility of people, such as parks and city squares [87], while old urban residential communities have lower mobility and longer stay times [88,89], so this variability may also be due to the different functional attributes of the sites [90].

In previous studies, it has been shown that shading facilities have a significant impact on activity patterns and behaviors in hot scenarios. It was found that in the 16 scenarios used in the experiment, using a landscape corridor, pavilion, or trees for shading in cooler weather resulted in a cooler perception pattern in the TPS of the residents. The use of arbors and gazebos with a dense canopy still had a high activity intention when the residents perceived the site as hot. The mediation between landscape configuration parameters in TPS, LQE, and activity willingness reveals that residents perceive that using these shading measures will give higher LQE due to the excellent microclimate conditions. In addition to the fact that trees are highly predictive of TPS and increase site activity intentions in hot weather, the mediated pathway shows an interesting phenomenon in which residents increase their activity intentions even in cool weather, because of the good microclimate conditions provided by trees. This is consistent with the practice of Huang and Zhou et al. [91]. They added vegetation and shade to a playground in Wuhan, which increased the number of occupants coming to the site by more than 80%, even in more comfortable weather, and increased the duration of activists on the site. In addition, the pavilion also had the same effect as the trees. At the same time, the increase in the willingness to move around the site by the corridor is more based on the residents’ perception that the presence of the corridor significantly improves the LQE of the environment. In terms of paving materials, there was no significant difference in TPS between the four paving materials when residents perceived the site as hotter, but the use of gray permeable tiles elicited a stronger willingness to move; while floor paving with colored plastic had a higher LQE, and its use in a long-term cooler space would better increase residents’ activity willingness. The scenes with concrete paving showed non-idealized TPSV, LQE, and activity willingness in both weather conditions. Light-colored paving materials have an inherent high albedo and emissivity due to their characteristic ability to keep surface temperatures low [92,93]; even in hot weather, light and dark materials maintain a temperature difference range between 5 and 11 °C [94], but the general perception in residents’ TPS that scenarios with outdoor tiles would be hotter presents the opposite to the reality, and the residents’ willingness to be active is not high. Previous research based on empirical studies found that using light-colored stone on floors or walls instead increased thermal stress through visualization, and found that the surface texture and color of the floor material is the main factor affecting subjective thermal perception [92]. Hence, the research in this paper reflects the possibility that TPS may be influenced by visualization and, thus, by the path of high albedo surfaces rather than the actual perceived temperature.

Looking at the four activity-type sites, similar to the findings of Jiaqi Niu and Jiangpeng Xiong et al. based on field surveys combined with numerical simulations, spaces with more tables and chairs tend to involve less intense activities. In contrast, areas involving activity facilities, even with unsatisfactory thermal environments, can attract frequent visits due to their supportive activity facilities [95]. While looking at the other three activity facilities, the children’s activity facilities reduced the intention to be active either from the mediated path of TPS or LQE, which may be related to the fact that the study sample in this paper is primarily residents aged 16 years and above. Studies have shown that children are more likely to be present in such spaces over a broader range of thermal conditions and that adults, as caregivers of children, are more concerned with temperature changes in the activity space than as users and enjoyers of such spaces [96]. In addition, we found that in hot weather, even without excessive shade facilities, residents are highly willing to be active in open spaces with seating. At the same time, the elderly do not respond quickly to the rise in temperature when they are sedentary in open spaces [97,98], which can be interpreted as a disadvantage for hot weather conditions, as it increases the probability of people being active but does not provide a comfortable thermal environment, so comfortable microclimate conditions should be provided in such open spaces to circumvent the thermal risks associated with the activity.

### 5.4. Advantages, Limitations, and Future Work

The novelty of this study is that it explores the relationship between TPS and activity intentions, and finds that age and SES lead to perceived bias in TPS. This finding complements previous studies on why older and lower economic residents are more likely to visit outdoor spaces for activities [75,99], i.e., part of the reason can be explained by the bias that has developed in TPS for destinations for multiple reasons. This study can enhance urban planners’ understanding of the factors and requirements related to thermal perception, usage patterns, and open space use for specific populations.

Over the past few decades, many scholars have realized that extrinsic perceptual predictions through physiological indicators are not immediately useful for urban design practice. These studied factors include the level of clothing [100], time spent outdoors [101], physiological changes in exposure to different temperatures, and weather conditions [102], as these physiological transients are often “transient” in nature and are independent of spatial context. In contrast, urban design interventions are inherently spatial, often quite persistent, and do not readily respond to “transient” conditions. This study reveals that the long-term experience of thermal perception is more stable than “transient” nature, and finds some landscape parameters that can mediate the TPS→activity willingness, i.e., what physical environment triggers what kind of perception, which can essentially remedy the previous dilemma about the difficulty of applying subjective thermal perception to urban design.

On the other hand, there are some limitations to this study. First, due to the survey sample, this study focuses on TPS among residents aged 16 and above, and does not represent well the group under 15 years old. Second, since the usage attributes of old urban residential community spaces are very different from other types of spaces, which may lead to partial bias in the study results, this leads to the fact that this study may not apply to all scenarios, so subsequent studies can be conducted for different spatial environments to provide more specific design solutions for different environments. In addition, the influence of the social/demographic characteristics of the participants on the mechanism implies that in-depth studies of specific groups should be conducted, and that different design strategies should be considered more comprehensively, especially at the age and at the SES level. Additionally, it should be noted that other factors affecting heat perception were not included in the study compared to the influencing factors discussed, such as sound, color, light intensity, etc. Therefore, follow-up studies should further explore these factors to establish a complete research system. Finally, TPS, as an experience of long-term thermal perception in the real world, combining real-world physiological thermal perception with TPS will better enrich our future research work, so the next step to consider how to develop a better experimental method in the real world is also the focus of future research work.

## 6. Conclusions

Over the past few decades, many scholars have studied how to increase the willingness to activate outdoor spaces. For example, these studies have found that landscape configuration parameters, such as lighting, signage, facility location, landscape design, maintenance frequency, and user distance from the destination, affect resident access and use [103,104,105,106]. Another important factor affecting the destinations used in this study is revealed: “TPS”. In this paper, we explore the differences in TPS among different populations and explore the TPS and the mediating paths that common landscape configuration elements in old residential communities form in the minds of residents. Equally important is that some practical conclusions can be drawn from our research about shaping public spaces in old residential communities for special populations to help develop designs and plans.

1. The research in this paper shows that the older age groups tend to perceive the site-specific TPS as cooler, which means that both groups are more likely to enter the space for activities even in hot conditions; to cater to the unique needs of the elderly who visit parks for TPS, it is recommended that planning and design should optimize cooling and thermal comfort capabilities, such as adding trees, water ponds, and shade facilities to their open spaces, along with design approaches that are effective in regulating microclimates to provide a more comfortable activity environment. For SES levels, while earlier studies have shown that less affluent and poorer residents use urban parks more seriously as thermal refuges, some essential and less costly landscape elements found in this study can be effective in increasing their willingness to move, such as replacing expensive pavilions and landscape corridors with more trees, and reducing the use of shrubs in community spaces in favor of easy-to-care-for grass.
2. In both groups of samples, no matter how the environment changes, we found that trees have formed cool inherent TPS in residents’ minds, and always have a positive relationship with AVQ and activity willingness. In addition, placing tree ponds for resting under trees can increase residents’ activity willingness, even in hot conditions. Increasing the proportion of grass in urban spaces and avoiding large areas of hard ground pavement can improve the TPS and LQE perceptions of space residents and increase activity willingness.
3. In recent years, light-colored materials with high reflectivity have been applied to the built environment in the construction of green cities, such as roofs, fences, floors, etc. However, in this study, it was found that light-colored materials may not be suitable for large-area use as floor coverings in urban spaces, and it may be an important limiting factor, especially in scenarios with high temperature and radiation, which can affect outdoor citizens’ willingness to move around in outdoor spaces.
4. The use of pavilions or trees in outdoor spaces that are chronically hot stimulates residents’ willingness to move more than the use of porches; in cities with chronically cool weather, the presence of landscape corridors can increase residents’ desire to move because of its increased LQE. However, pavilions may become a more viable solution for narrow roadsides in outdoor spaces, where growing space is insufficient and soil volume is limited.

While determining the effect of TPS on the willingness to be active, this study reminds relevant practitioners that they should not only consider physiological thermal indicators, but the design of public spaces should respect the needs of occupants with different socio-demographic backgrounds. In addition, more attention should be paid to the environment and amenities, such as trees, activity facilities, and seating, which significantly impact TPS, to improve the attractiveness and frequency of use of community spaces.

## Figures and Tables

**Table 1:** Summary table of the proportional distribution of community public space feature codes.

Environmental Characteristics of Old Residential Communities (*n* = 921)	Coding Scores and Percentages	Environmental Characteristics of Old Residential Communities (*n* = 921)	Coding Scores and Percentages
Site rest features		Shading facility features	
Tree pond with plants	(621) 67.40%	trees	(521) 56.5%
Wooden benches	(211) 22.9%	Landscape corridor	(119) 12.9%
Single stone bench	(41) 4.4%	Pavilion	(63) 6.8%
Table and chair sets	(36) 3.9%	No shading facilities	(140) 15.2%
Others	(12) 1.3%	Others	(25) 2.7%
Common site activity type characteristics		Common site activity type characteristics	
Concrete pavement	(421) 45.7%	Leisure type venues	(435) 47.2%
Permeable brick pavement	(312) 33.8%	Children’s activity type venues	(110) 11.9%
Tile pavement	(98) 10.6%	Fitness activity type venue	(320) 34.70%
Plastic cement pavement	(39) 4.2%	Sport Type Venue	(37) 4%
Others	(51) 5.5%	Others	(19) 2%
Architectural wall features			
Concrete walls	(351) 38.1%		
Painting with Coatings	(269) 29.2%		
Accompanied by climbing plants	(98) 10.6%		
Graffiti	(65) 7%		
Others	(108) 11.7%		

**Table 2:** Measurement scale of TPS, LQE, and activity willingness.

Variables	Questions and Measurement Scale		
TPSV	It would be very cold to be in this scene.	1.___2.___3.___4.___5.___6.___7.___	It would be very hot to be in this scene.
LQE	This scene is not beautiful at all	1.___2.___3.___4.___5.___6.___7.___	This scene is very beautiful
	This scene is very boring	1.___2.___3.___4.___5.___6.___7.___	This scene is very interesting
	The environment here does not attractive to me at all.	1.___2.___3.___4.___5.___6.___7.___	The environment here is very attractive to me
	The environment here doesn’t appeal to me at all.	1.___2.___3.___4.___5.___6.___7.___	The environment here is very appealing to me
Willingness to be actively involved	I do not want to do activities in this scene.	1.___2.___3.___4.___5.___6.___7.___	I want to do activities in this scene

**Table 3:** Coding and classification of demographic variables and related data.

Variables to Be Recoded and Regrouped	Ordinal Groups		
	0	1	2
GROUP1-TPSV	Score < 4	/	Score > 4
GROUP2-TPSV	Score < 4	/	Score > 4
GENDER	Male	/	Female
AGE	<25 years	26~35 years	>36 years
Access Frequency	<Twice a week	/	>Three times a week
Activity Duration	<30 min	31~60 min	>60 min
Living Area	Southern Cities	/	Northern Cities
Years of residence	<5 years	/	>5 years

**Table 4:** Artificial Intelligence Convolutional Neural Network (CNN) Major Landscape Elements Recognition Output Data.

Filename	Buildings	Sky	Ground	Trees	Tree Ponds	Shrubs	Grass	Pavilion	Landscape Corridor	Tables and Chairs	Children’s Facilities	Leisure Facilities	Sports Facilities
A1	0.352	0.082	0.342	0.000	0.044	0.061	0.000	0.000	0.069	0.000	0.000	0.000	0.000
A2	0.354	0.081	0.209	0.000	0.045	0.062	0.098	0.000	0.060	0.000	0.000	0.000	0.000
A3	0.163	0.061	0.163	0.274	0.046	0.000	0.166	0.000	0.036	0.000	0.000	0.000	0.000
A4	0.163	0.062	0.101	0.281	0.044	0.087	0.245	0.000	0.024	0.000	0.000	0.000	0.000
B1	0.263	0.001	0.321	0.269	0.044	0.000	0.000	0.000	0.036	0.000	0.000	0.010	0.000
B2	0.183	0.082	0.329	0.269	0.043	0.000	0.000	0.000	0.037	0.000	0.000	0.010	0.000
B3	0.154	0.133	0.321	0.270	0.045	0.000	0.000	0.000	0.033	0.000	0.000	0.010	0.000
B4	0.071	0.181	0.322	0.271	0.043	0.000	0.000	0.000	0.036	0.022	0.000	0.010	0.000
C1	0.281	0.082	0.361	0.133	0.022	0.011	0.021	0.000	0.000	0.022	0.000	0.000	0.000
C2	0.208	0.083	0.178	0.134	0.021	0.010	0.022	0.000	0.232	0.022	0.000	0.000	0.000
C3	0.126	0.032	0.165	0.133	0.020	0.009	0.022	0.335	0.000	0.022	0.000	0.000	0.000
C4	0.101	0.043	0.271	0.381	0.081	0.010	0.022	0.000	0.000	0.022	0.000	0.000	0.000
D1	0.171	0.082	0.351	0.241	0.044	0.000	0.000	0.000	0.031	0.000	0.000	0.010	0.000
D2	0.163	0.083	0.351	0.241	0.046	0.000	0.000	0.000	0.032	0.000	0.000	0.010	0.000
D3	0.165	0.082	0.351	0.243	0.044	0.000	0.000	0.000	0.031	0.000	0.000	0.010	0.000
D4	0.163	0.081	0.351	0.241	0.041	0.000	0.000	0.000	0.034	0.000	0.000	0.010	0.000
E1	0.191	0.081	0.355	0.198	0.012	0.000	0.000	0.000	0.032	0.000	0.000	0.041	0.000
E2	0.191	0.082	0.355	0.198	0.011	0.000	0.000	0.000	0.033	0.000	0.078	0.000	0.000
E3	0.192	0.081	0.353	0.198	0.012	0.000	0.000	0.000	0.033	0.089	0.000	0.000	0.000
E4	0.193	0.081	0.351	0.198	0.011	0.000	0.000	0.000	0.036	0.011	0.000	0.000	0.070

**Table 5:** Summary statistics of participants’ background information and the results of the one-way ANOVA with TPS.

Demographic Characteristics		Group 1 (*n* = 1773)	F/P	Group 2 (*n* = 1662)	F/P
Gender	Male	855 (48.2%)	F = 0.337 *p* = 0.561	845 (50.8%)	F = 0.006 *p* = 0.936
	Female	918 (51.8%)		817 (49.2%)	
Age group	<15 years	43 (2.4%)	F = 51.143 *p* < 0.001	159 (9.5%)	F = 21.419 *p* < 0.001
	16~25 years	588 (33.2%)		501 (30.1%)	
	26~35 years	490 (27.6%)		485 (29.2%)	
	36~45 years	302 (17%)		281 (16.9%)	
	>45 years	350 (19.7%)		236 (14.2%)	
Years of residence	<1 years	278 (15.7%)	F = 1.666 *p* = 0.172	208 (12.5%)	F = 1.031 *p* < 0.378
	1~5 years	546 (30.8%)		411 (24.7%)	
	5~10 years	582 (32.8%)		607 (36.5%)	
	>10 years	367 (20.7%)		436 (26.2%)	
Occupation Type	Retired	254 (14.3%)	F = 51.127 *p* < 0.001	55 (3.3%)	F = 29.864 *p* < 0.001
	Student	537 (30.3%)		280 (16.8%)	
	manual labor	326 (18.4%)		652 (39.2%)	
	intellectual labor	656 (37%)		675 (40.6%)	
Educational Background	primary school	232 (13.1%)	F = 31.169 *p* < 0.001	76 (4.6%)	F = 24.539 *p* < 0.001
	middle school	183 (10.3%)		211 (12.7%)	
	high school	306 (17.3%)		199 (12.0%)	
	Bachelor’s degree	778 (43.9%)		857 (51.6%)	
	Master’s degree and above	274 (15.5%)		319 (19.2%)	
Monthly income level (RMB)	<3000	650 (36.7%)	F = 18.233 *p* < 0.001	310 (18.7%)	F = 19.459 *p* < 0.001
	3000~5000	336 (19%)		427 (25.7%)	
	5000~7000	253 (14.3%)		418 (25.2%)	
	7000~9000	236 (13.3%)		258 (15.5%)	
	>9000	298 (16.8%)		247 (14.9%)	
Frequency of Visit	Rarely used	296 (16.7%)	F = 2.317 *p* = 0.074	381 (22.9%)	F = 0.233 *p* = 0.873
	1~2 times a week	608 (34.3%)		508 (30.6%)	
	3~4 times a week	550 (31%)		524 (31.5%)	
	More than 5 times a week	319 (18%)		249 (15.0%)	
Activity Duration	<15 min	309 (17.4%)	F = 2.094 *p* = 0.079	387 (23.3%)	F = 1.064 *p* = 0.373
	16~30 min	429 (24.2%)		437 (26.3%)	
	31~60 min	483 (27.2%)		436 (26.2%)	
	60~90 min	382 (21.5%)		202 (12.2%)	
	>90 min	170 (9.6%)		200 (12.0%)	
Living Area	Southern Cities	1035 (58.4%)	F = 2.749 *p* = 0.097	807 (48.6%)	F = 0.109 *p* = 0.742
	Northern Cities	738 (41.6%)		855 (51.4%)	

**Table 6:** Dominance ratios and 95% confidence intervals for binary logistic LR stepwise regression analysis of the relationship between demographic variables and TPSV (Note: *** *p* ≤ 0.001, ** *p* ≤ 0.01).

		Group 1		Group 2	
		TPSV < 4 (Ref. TPSV > 4)		TPSV < 4 (Ref. TPSV > 4)	
		Odds Ration	*p*	Odds Ration	*p*
		(95% CI)		(95% CI)	
Age	Ref. (<25 years)				
	26~35 years	1.081 (0.765, 1.528)	0.657	0.329 (0.218, 0.498)	<0.001 ***
	>35 years	0.266 (0.200, 0.354)	<0.001 ***	0.188 (0.128, 0.275)	<0.001 ***
	Ref (>35 years)				
	26~35 years	4.062 (2.965, 5.565)	<0.001 ***	1.754 (1.245, 2.471)	<0.01 **
Socioeconomic Status	SES	1.581 (1.383, 1.807)	<0.001 ***	2.349 (2.005, 2.752)	<0.001 ***
	Pseudo-R2	0.113		0.112	
	Hosmer-Lemeshow	0.817		0.521	

**Table 7:** TPSV means and ANOVA analysis and LSD post-hoc test for different landscape parameters and feature types in Group 1 (Note: *** *p* ≤ 0.001, ** *p* ≤ 0.01).

Landscape Parameters Configuration (Group 1)	1	2	3	4	Overall	F-Stats	*p*-Value	Post-Hoc LSD
A-Green Dimension (*n* = 402)								
TPSV:	5.41	4.84	3.89	3.41	4.42	47.788	*p* < 0.001 ***	A1 to A2,A1 to A3,A1 to A4, A2 to A3,A2 to A4,A3 to A4
LQE:	3.33	3.99	4.84	5.58	4.39	68.857	*p* < 0.001 ***	A1 to A2,A1 to A3,A1 to A4, A2 to A3,A2 to A4,A3 to A4
Activity willingness:	3.10	3.58	4.93	3.90	3.91	32.889	*p* < 0.001 ***	A1 to A2,A1 to A3,A1 to A4, A2 to A3,A2 to A4,A3 to A4
B-sky Dimension (*n* = 360)								
TPSV:	4.11	4.95	5.22	5.25	4.86	16.016	*p* < 0.001 ***	B1 to B2,B1 to B3,B1 to B4, B2 to B4
LQE:	3.86	4.46	5.20	5.11	4.64	27.533	*p* < 0.001 ***	B1 to B2,B1 to B3,B1 to B4, B2 to B3,B2 to B4
Activity willingness:	4.36	4.53	5.09	4.96	4.73	7.515	*p* < 0.001 ***	B1 to B3,B1 to B4,B2 to B3, B2 to B4
C-Shade type dimension (*n* = 327)								
TPSV:	4.68	3.84	4.24	3.68	4.11	6.410	*p* < 0.001 ***	C1 to C2,C1 to C4,C3 to C4
LQE:	4.02	4.63	5.02	5.36	4.71	13.19	*p* < 0.001 ***	C1 to C2,C1 to C3,C1 to C4, C2 to C4
Activity willingness:	4.11	4.78	5.20	5.16	4.81	8.844	*p* < 0.001 ***	C1 to C2,C1 to C3,C1 to C4
D-Pavement type dimension (*n* = 327)								
TPSV:	5.01	4.56	5.26	4.60	4.86	4.236	*p* < 0.01 **	D1 to D4,D2 to D3,D3 to D4
LQE:	4.48	4.14	3.49	4.72	4.21	10.411	*p* < 0.001 ***	D1 to D3,D2 to D3,D2 to D4, D3 to D4
Activity willingness:	4.14	4.44	3.74	4.30	4.16	3.101	*p* < 0.01 **	D2 to D3,D3 to D4
E-Activity Type Dimension (*n* = 357)								
TPSV:	4.72	5.31	4.89	5.04	5.29	4.559	*p* < 0.01 **	E1 to E2,E1 to E4,E2 to E3, E3 to E4
LQE:	4.28	3.94	4.24	4.73	4.30	6.008	*p* < 0.01 **	E1 to E4,E2 to E4,E3 to E4
Activity willingness:	4.18	4.06	4.09	4.79	4.27	4.821	*p* < 0.01 **	E1 to E4,E2 to E4,E3 to E4

**Table 8:** TPSV means and ANOVA analysis and LSD post hoc test for different landscape parameters and feature types in Group 2 (Note: *** *p* ≤ 0.001, ** *p* ≤ 0.01).

Landscape Parameters Configuration (Group 2)	1	2	3	4	Overall	F-Stats	*p*-Value	Post-Hoc LSD
A-Green Dimension (*n* = 329)								
TPSV:	5.68	4.98	4.58	3.92	4.79	24.346	*p* < 0.001 ***	A1 to A2,A1 to A3,A1 to A4, A2 to A4,A3 to A4
LQE:	2.93	3.21	4.59	5.58	5.03	53.582	*p* < 0.001 ***	A1 to A3,A1 to A4,A2 to A3, A2 to A4,A3 to A4
Activity willingness:	3.19	3.26	4.80	5.45	4.17	53.662	*p* < 0.001 ***	A1 to A3,A1 to A4,A2 to A3, A2 to A4,A3 to A4
B-sky Dimension (*n* = 333)								
TPSV:	4.05	4.99	5.20	5.79	5.01	18.972	*p* < 0.001 ***	B1 to B2,B1 to B3,B1 to B4, B2 to B4,B3 to B4
LQE:	4.65	4.42	4.14	3.69	4.22	6.024	*p* < 0.01 **	B1 to B3,B1 to B4,B2 to B4
Activity willingness:	4.60	4.45	3.75	3.44	4.06	11.033	*p* < 0.001 ***	B1 to B3,B1 to B4,B2 to B3, B2 to B4
C-Shade type dimension (*n* = 332)								
TPSV:	5.37	4.83	4.25	4.20	4.67	10.762	*p* < 0.001 ***	C1 to C2,C1 to C3,C1 to C4, C2 to C3,C2 to C4
LQE:	3.78	4.35	4.59	5.05	4.44	13.19	*p* < 0.001 ***	C1 to C2,C1 to C3,C1 to C4, C2 to C4,C3 to C4
Activity willingness:	3.24	3.83	4.90	5.65	4.43	45.451	*p* < 0.001 ***	C1 to C2,C1 to C3,C1 to C4, C2 to C3,C2 to C4,C3 to C4
D-Pavement type dimension (*n* = 333)								
TPSV:	5.24	4.98	5.34	5.11	5.17	1.045	*p* = 0.373	
LQE:	4.21	4.46	3.56	4.60	4.21	10.794	*p* < 0.001 ***	D1 to D3,D1 to D4,D2 to D3, D3 to D4
Activity willingness:	3.95	4.61	3.64	3.93	4.04	6.279	*p* < 0.001 ***	D1 to D2,D2 to D3,D2 to D4
E-Activity Type Dimension (*n* = 357)								
TPSV:	5.05	5.29	5.07	5.36	5.19	1.101	*p* = 0.349	
LQE:	3.92	3.51	3.67	3.77	3.72	1.682	*p* = 0.171	
Activity willingness:	3.24	3.28	3.36	3.13	3.25	0.315	*p* = 0.814	

**Table 9:** Regression coefficients (%) of landscape parameters with TSPV from Group 1 stepwise multiple linear regression analysis (b) (Note: *** *p* ≤ 0.001, ** *p* ≤ 0.01, * *p* ≤ 0.05).

Group 1		TPSV	LQE	Activity Willingness
	Adj.R^2^	0.165	0.211	0.158
Building	R^2^_partial_	−0.024	0.090	0.063
	b	−0.056	−0.197 **	−0.169 **
Sky	R^2^_partial_	0.168	0.108	0.124
	b	0.165 ***	0.129 ***	0.179 ***
Ground	R^2^_partial_	0.241	−0.131	−0.162
	b	0.256 ***	−0.171 ***	−0.124 ***
Trees	R^2^_partial_	−0.159	0.271	0.093
	b	−0.157 ***	0.338 ***	0.251 ***
Tree ponds	R^2^_partial_	0.071	−0.003	0.111
	b	0.077 **	−0.004	0.131 ***
Shrubs	R^2^_partial_	−0.023	0.101	−0.163
	b	−0.039	0.145 ***	−0.278 ***
Grass	R^2^_partial_	−0.026	0.032	−0.046
	b	−0.036	0.022	−0.076
Pavilion	R^2^_partial_	0.040	0.047	0.117
	b	0.047	0.059	0.115 ***
Landscape corridor	R^2^_partial_	−0.056	−0.024	0.037
	b	−0.058 *	−0.027	0.047
Children’s facilities	R^2^_partial_	0.080	−0.062	0.007
	b	0.079 **	−0.058 *	0.007
Leisure facilities	R^2^_partial_	−0.003	−0.008	0.020
	b	−0.003	−0.007	0.021
Sports facilities	R^2^_partial_	0.080	−0.062	0.007
	b	0.079 **	−0.058 *	0.007

**Table 10:** Regression coefficients (%) of landscape parameters with TSPV from Group 2 stepwise multiple linear regression analysis (b) (Note: *** *p* ≤ 0.001, * *p* ≤ 0.05).

Group 2		TPSV	LQE	Activity Willingness
	Adj.R^2^	0.117	0.161	0.108
Building	R^2^_partial_	−0.019	0.010	−0.007
	b	−0.040	0.020	−0.023
Sky	R^2^_partial_	0.215	−0.127	−0.155
	b	0.216 ***	−0.123 ***	−0.148 ***
Ground	R^2^_partial_	0.167	−0.220	−0.113
	b	0.166 ***	−0.158 ***	−0.136 ***
Trees	R^2^_partial_	−0.155	0.318	0.204
	b	−0.134 ***	0.308 ***	0.320 ***
Tree ponds	R^2^_partial_	−0.052	0.009	0.070
	b	−0.072	0.012	0.115 *
Shrubs	R^2^_partial_	−0.029	−0.012	−0.018
	b	−0.038	−0.015	−0.051
Grass	R^2^_partial_	−0.027	0.006	0.136
	b	−0.028	0.006	0.155 ***
Pavilion	R^2^_partial_	0.021	0.018	0.035
	b	0.022	0.017	0.046
Landscape corridor	R^2^_partial_	0.004	−0.002	−0.030
	b	0.005	−0.002	−0.031
Children’s facilities	R^2^_partial_	0.038	−0.023	−0.029
	b	0.036	−0.021	−0.028
Leisure facilities	R^2^_partial_	0.020	−0.062	0.003
	b	0.019	−0.059 *	0.002
Sports facilities	R^2^_partial_	0.011	0.012	0.098
	b	0.010	0.012	0.109 ***

**Table 11:** Regression coefficients of the two groups on LQE on activity willingness (note: R^2^_partial_ is the correlation coefficient after controlling for other variables, *** *p* ≤ 0.001, ** *p* ≤ 0.01).

	LQE	Activity Willingness
Group1-TPSV(models with a single a single predictor)	b = −0.279 ***	b = −0.226 ***
	R^2^ = 0.077	R^2^ = 0.050
Group1-TPSV (models with Control-related variables)	b = −0.160 ***	b = −0.069 **
	R^2^_partial_ = −0.190	R^2^_partial_ = −0.081
Group2-TPSV(models with a single a single predictor)	b = −0.643 ***	b = −0.646 ***
	R^2^ = 0.413	R^2^ = −0.417
Group2-TPSV (models with Control-related variables)	b = −0.467 ***	b = −0.472 ***
	R^2^_partial_ = −0.436	R^2^_partial_ = −0.441

**Table 12:** The mediating role of LQE between TPS and activity willingness in two groups.

Mediator	Indirect Effect	SE	Standardized Indirect Effect	Proportion Mediated (%)	95% CI (Bootstrap *n* = 5000)	
					LL	UL
Group1-TPSV→LQE→Activity willingness	−0.1595	0.0165	−0.1572	69.5%	−0.1936	−0.1279
Group2-TPSV→LQE→Activity willingness	−0.1770	0.0182	−0.1610	25.2%	−0.2139	−0.1420

**Table 13:** Mediation of landscape configuration parameters among TPSV, LQE, and activity willingness (Note: Bolded fonts are significant paths of landscape configuration mediation).

Intermediate Variables	Group 1			Group 2		
Landscape parameters (%)	TPSV→Landscape parameters→Activity Willingness	TPSV→Landscape parameters→LQE	LQE→Landscape parameters→Activity Willingness	TPSV→Landscape parameters→Activity Willingness	TPSV→Landscape parameters→LQE	LQE→Landscape parameters→Activity Willingness
Building	**IE = 0.024**	**IE = −0.039**	**IE = −0.050**	**IE = 0.014**	**IE = −0.013**	**IE = 0.026**
	**IEP = 0.123**	**IEP = 0.155**	**IEP = 0.076**	**IEP = 0.019**	**IEP = 0.021**	**IEP = 0.041**
Sky	**IE = 0.022**	**IE = 0.03**	IE = −0.001	**IE = −0.022**	IE = −0.004	**IE = 0.029**
	**IEP = −0.010**	**IEP = −0.141**	IEP = −0.003	**IEP = 0.032**	IEP = 0.006	**IEP = 0.045**
Ground	IE = −0.002	**IE = −0.050**	**IE = −0.017**	**IE = −0.031**	IE = −0.008	**IE = 0.035**
	IEP = 0.008	**IEP = 0.199**	**IEP = −0.025**	**IEP = 0.045**	IEP = −0.014	**IEP = 0.054**
Trees	**IE = −0.050**	**IE = −0.045**	**IE = 0.055**	**IE = −0.167**	**IE = −0.027**	**IE = 0.042**
	**IEP = 0.216**	**IEP = 0.178**	**IEP = 0.084**	**IEP = 0.121**	**IEP = 0.045**	**IEP = 0.065**
Tree ponds	IE = −0.003	**IE = −0.008**	IE = −0.004	**IE = −0.022**	**IE = −0.008**	**IE = 0.034**
	IEP = 0.012	**IEP = 0.032**	IEP = −0.006	**IEP = 0.032**	**IEP = 0.014**	**IEP = 0.053**
Shrubs	IE = −0.003	IE = −0.001	**IE = 0.029**	IE = 0.001	IE = 0.004	**IE = −0.007**
	IEP = 0.012	IEP = 0.003	**IEP = 0.044**	IEP = −0.001	IEP = −0.006	**IEP = −0.011**
Grass	**IE = 0.0235**	**IE = −0.028**	**IE = −0.040**	**IE = −0.018**	IE = −0.003	**IE = 0.019**
	**IEP = 0.102**	**IEP = 0.113**	**IEP = −0.058**	**IEP = 0.024**	IEP = 0.005	**IEP = 0.030**
Pavilion	**IE = −0.014**	**IE = 0.005**	IE = 0.000	**IE = −0.0683**	IE = −0.0001	**IE = 0.007**
	**IEP = 0.063**	**IEP = 0.018**	IEP = 0.000	**IEP = 0.0973**	IEP = −0.0001	**IEP = 0.012**
Landscape corridor	IE = 0.004	**IE = 0.006**	IE = −0.001	IE = −0.002	IE = −0.001	IE = 0.003
	IEP = −0.016	**IEP = −0.233**	IEP = −0.001	IEP = −0.003	IEP = 0.001	IEP = 0.005
children’s facilities	IE = −0.000	**IE = −0.006**	IE = 0.000	**IE = −0.036**	**IE = −0.003**	**IE = 0.005**
	IEP = 0.001	**IEP = 0.029**	IEP = 0.000	**IEP = 0.0509**	**IEP = 0.0496**	**IEP = 0.008**
Leisure facilities	IE = 0.002	IE = −0.000	IE = −0.001	**IE = −0.010**	IE = 0.001	IE = 0.006
	IEP = −0.008	IEP = 0.000	IEP = −0.001	**IEP = 0.014**	IEP = −0.001	IEP = 0.010
Sports facilities	**IE = 0.009**	**IE = 0.006**	IE = 0.000	IE = 0.005	IE = −0.000	**IE = 0.001**
	**IEP = 0.038**	**IEP = 0.024**	IEP = 0.000	IEP = 0.008	IEP = −0.000	**IEP = 0.009**

## Data Availability

The dataset generated and analyzed in this study is not publicly available; however, the dataset is available from the corresponding author upon reasonable request.
